# Structure of the N-terminal domain of the protein Expansion: an ‘Expansion’ to the Smad MH2 fold

**DOI:** 10.1107/S1399004715001443

**Published:** 2015-03-26

**Authors:** Mads Beich-Frandsen, Eric Aragón, Marta Llimargas, Jordi Benach, Antoni Riera, Joan Pous, Maria J. Macias

**Affiliations:** a Institute for Research in Biomedicine (IRB Barcelona), Baldiri Reixac 10, 08028 Barcelona, Spain; b Institut de Biologia Molecular de Barcelona, IBMB–CSIC, Baldiri Reixac 10, 08028 Barcelona, Spain; c ALBA Synchrotron, BP 1413, km 3.3, Cerdanyola del Vallès, Spain; d Departament de Química Orgànica, Universitat de Barcelona, Martí i Franqués 1-11, 08028 Barcelona, Spain; e Platform of Crystallography IBMB–CSIC, Baldiri Reixac 10, 08028 Barcelona, Spain; f Catalan Institution for Research and Advanced Studies (ICREA), Passeig Lluís Companys 23, 08010 Barcelona, Spain

**Keywords:** Expansion protein, Smad homology domain 2, MH2 domain, Smad/FHA protein superfamily, protein–protein interaction, *phenix.mr_rosetta*

## Abstract

Gene-expression changes observed in *Drosophila* embryos after inducing the transcription factor Tramtrack led to the identification of the protein Expansion. Expansion contains an N-terminal domain similar in sequence to the MH2 domain characteristic of Smad proteins, which are the central mediators of the effects of the TGF-β signalling pathway. Apart from Smads and Expansion, no other type of protein belonging to the known kingdoms of life contains MH2 domains. To compare the Expansion and Smad MH2 domains, the crystal structure of the Expansion domain was determined at 1.6 Å resolution, the first structure of a non-Smad MH2 domain to be characterized to date. The structure displays the main features of the canonical MH2 fold with two main differences: the addition of an α-helical region and the remodelling of a protein-interaction site that is conserved in the MH2 domain of Smads. Owing to these differences, to the new domain was referred to as Nα-MH2. Despite the presence of the Nα-MH2 domain, Expansion does not participate in TGF-β signalling; instead, it is required for other activities specific to the protostome phyla. Based on the structural similarities to the MH2 fold, it is proposed that the Nα-MH2 domain should be classified as a new member of the Smad/FHA superfamily.

## Abstract 2

Expansion is a modular protein that is conserved in protostomes. The first structure of the N-terminal domain of Expansion has been determined at 1.6 Å resolution and the new Nα-MH2 domain was found to belong to the Smad/FHA superfamily of structures.

## 1. Introduction   

The *Drosophila* transcription factor Tramtrack (Ttk) is involved in a wide range of processes during development of the tracheal system. The analysis of gene-expression changes in *Drosophila* embryos after inducing Ttk loss of function and gain of function enabled the identification of the *Expansion* gene (CG13188; *Exp*). A search for similar proteins to Expansion led to the identification of CG13183 (recently renamed Rebuf; Reb; Rotstein *et al.*, 2011 ▶). These two proteins share 56% amino-acid similarity that is concentrated in the N-terminal part of the sequence. Expansion and Rebuf (Exp/Reb) proteins have been identified in several *Drosophila* species, other insects and arthropods (Iordanou *et al.*, 2014 ▶). They are annotated in the NCBI database as modular proteins containing an N-terminal MH2 domain and a variable C-terminal region which does not present sequence similarity to other characterized domains. The sequence similarity to the MH2 domain is quite remarkable since these domains were believed to be exclusively present in Smad proteins, which are the main players in the TGF-β signalling pathway in metazoans (Massagué, 2012 ▶). Smad proteins comprise two conserved domains separated by a linker that does not adopt a defined tertiary structure. The N-terminal (MH1) domain binds to DNA sites in promoters, while the linker and the C-terminal (MH2) domain are the protein-interaction sites. As mediators and regulators of cytokine signalling, Smads are involved in many cellular processes from cell homeostasis to differentiation, division and cell death (Massagué *et al.*, 2005 ▶).

The presence of an MH2 domain in Exp/Reb proteins has been used as a hallmark to classify them as Smad-like proteins (Iordanou *et al.*, 2014 ▶). However, the sequence identity of the Exp/Reb proteins to the Smads is very low and is restricted to the MH2 domain. Furthermore, the differences are not only at the sequence level but also in the localization of the domain: in the N-terminal part in Exp/Reb in contrast to a C-terminal position in Smads. Secondary-structure predictions indicate that the Exp/Reb MH2 domain might contain additional elements of secondary structure preceding the MH2 fold. All of these characteristics suggest that Exp/Reb might constitute a new family of proteins that share the presence of a divergent MH2 domain with the Smads.

To clarify this issue and prompted by the similarities and differences between the Exp/Reb and Smads proteins, we set out to investigate the presence of the Exp/Reb proteins in metazoans and to characterize the structure of this new MH2 domain. Our results reveal that Exp/Reb proteins are restricted to protostomes, whereas Smads are highly conserved in both protostomes and deuterostomes. Regarding the structure, the crystal structure of this domain has been determined at 1.6 Å resolution and represents the first structure of an MH2 domain to be defined outside the Smad family of proteins. Although the structure displays the main features of the MH2 fold, it contains an additional α-helical region that covers the concave site of the MH2 domain and defines the specific structure of the Exp/Reb MH2 domain. Based on this observation, we refer to the Exp/Reb MH2 domain as an Nα-MH2 domain, a new member of the FHA/Smad superfamily of MH2 domains.

A characteristic of activated Smad proteins is the formation of quaternary structures through interactions of their MH2 domains (Shi & Massagué, 2003 ▶). Even if Exp/Reb proteins are different from Smads, the presence of the Nα-MH2 domain led us to hypothesize that Exp/Reb proteins could perhaps also modulate TGF-β signalling in protostomes through the formation of heterotrimers using the Nα-MH2 domain as a binding partner for Smads. The functional and structural studies presented here support the nonparticipation of Expansion in the canonical TGF-β signalling pathway. Analysis of the Nα-MH2 structure and its comparison with those of Smad proteins provides the basis for understanding the binding differences. Our data are in agreement with the results reported in the literature indicating that Exp/Reb proteins are required for specific activities in protostomes, regulating receptor tyrosine kinase signalling to control terminal branch size and morphology (Iordanou *et al.*, 2014 ▶).

## 2. Experimental procedures   

### 2.1. Sequence alignment and secondary-structure prediction   

Sequences corresponding to Expansion proteins were retrieved from the Ensembl Metazoa database (http://metazoa.ensembl.org) with *PSI-BLAST* (Altschul *et al.*, 1990 ▶), using the *D. melanogaster* Expansion protein (CG13188) as the query. Multiple sequence alignments with the query and the target proteins were generated with *MAFFT* v.7.164b using the iterative L-INS-i method (parameters: Blosum62 scoring matrix and gap-opening penalty set to 1.5).

Conserved residues were highlighted using the *BoxShade* server v.3.21 (http://sourceforge.net/projects/boxshade/) written by K. Hofmann and M. Baron. A graphical representation of secondary structure was added to the alignment using the *ESPript* server (Robert & Gouet, 2014 ▶).

Prediction of secondary-structure content was performed using the online version of *NetSurfP* (http://www.cbs.dtu.dk/services/NetSurfP/; Petersen *et al.*, 2009 ▶).

### 2.2. Cloning of the Expansion Nα-MH2 domain   

Constructs for the Expansion domain were cloned into the pETM-11 expression vector (EMBL) by means of ligation-independent cloning. The initial construct used for NMR screening and preliminary crystallization trials consisted of a fragment comprised of residues 29–240. We also prepared a second construct including an N-terminal extension (residues 3–240), which was used for structural studies.

Inserts were obtained by PCR using the appropriate primers and the *D. melanogaster* Expansion cDNA (CG13188, isoform B) as a template. PCR amplification was carried out using the standard PCR Master Mix with 0.02 unit µl^−1^ *Taq* DNA polymerase (Thermo Scientific), 1 ng µl^−1^ template DNA and 0.5 µ*M* of each primer, using an annealing temperature of 321 K for the first ten rounds and 341 K for the last 30 rounds. After subsequent purification of the reaction product using the GeneJET PCR purification kit (Fermentas), ligation-independent cloning was performed in the presence of 6 µg µl^−1^ RecA DNA recombinase in the recommended buffer (New England Biolabs) at 310 K for 15 min. The full recombination reaction was then used for transformation in *E. coli* DH5α and positive clones were selected on kanamycin agar plates (50 µg ml^−1^). Positives clones were verified by DNA sequencing and subsequently transformed into the expression strain *E. coli* BL21 (DE3) Rosetta (Invitrogen), selecting positive clones on kanamycin (50 µg ml^−1^) + chloramphenicol (34 µg ml^−1^) agar plates.

### 2.3. Protein expression and purification   

Cultures were grown at 310 K until an OD of ∼0.6 was reached; the temperature was then lowered to 293 K and protein expression was induced by the addition of 1 m*M* isopropyl β-d-1-thiogalactopyranoside (IPTG). Expression took place for 12–15 h and the cells were harvested by centrifugation (4000*g*, 15 min). Labelled samples for NMR were prepared in a similar manner using minimal medium (M9) enriched with ^15^NH_4_Cl.

The cells were washed in TBS buffer and 5 g of cells were resuspended at 277 K in 10 ml buffer consisting of 50 m*M* Tris–HCl pH 7.2, 500 m*M* NaCl, 1 m*M* phenylmethylsulfonylfluoride (PMSF), 0.1 m*M* β-mercaptoethanol (BME), 0.1 m*M* EDTA supplemented with 0.1 mg ml^−1^ lysozyme, 0.25 mg ml^−1^ DNaseI, 25 m*M* MgCl_2_, 0.01 mg ml^−1^ RNaseA for lysis. Lysis was performed in a pressurized cell homogenizer at 277 K and the crude solution was incubated for 20 min on ice. The soluble fraction was then isolated by centrifugation (45 000*g*, 20 min, 277 K).

Immobilized metal-ion affinity chromatography (IMAC) was performed on an ÄKTA FPLC system at room temperature using a prepacked 1 ml His-tag column (GE Healthcare). The domain of interest was purified using buffer consisting of 50 m*M* Tris–HCl pH 7.2, 500 m*M* NaCl, 0.1 m*M* BME, 0.1 m*M* EDTA and eluted with a gradient to the same buffer freshly supplemented with 500 m*M* imidazole (50 m*M* Tris–HCl pH 7.2, 500 m*M* NaCl, 0.1 m*M* BME, 0.1 m*M* EDTA, 500 m*M* imidazole). Peak fractions were isolated and diluted with buffer (50 m*M* Tris–HCl pH 7.2, 200 m*M* NaCl, 0.1 m*M* BME) to lower the salt and imidazole concentrations for enzymatic digestion. Subsequently, the N-terminal 6×His tag was removed by overnight digestion at 277 K with *Tobacco etch virus* (TEV) protease and the digested protein was repurified by IMAC as described above. Size-exclusion chromatography (SEC) was performed on a Superdex 200 10/300 column using buffer consisting of 50 m*M* Tris–HCl pH 7.2, 200 m*M* NaCl, 0.1 m*M* BME and the peak fractions were collected and concentrated to ∼10 mg ml^−1^ using centrifugal filters (Amicon).

### 2.4. *Drosophila* strains and genetics   

The fly strains used are described in FlyBase. Df(2R)ED2247 and Df(2R)BSC879 uncover CG13188 and CG13183 and were used in transheterozygous conditions to analyze the absence of both genes (CG13188+CG13183 mutants). The transgenes used were P(TRiP.HMS01445)attP2 (UAS13188RNAi), P(TRiP.HMS01444)attP2 (UAS13183RNAi), P(TRiP.JF02218)attP2 (UASMedRNAi), UASDad and UASTkvCA.

For overexpression experiments, we used the Gal4/UAS system (Brand & Perrimon, 1993 ▶). We used the breathlessGal4 (btlGal4) driver, which drives expression in all tracheal cells, and the nubbinGal4 (nubGal4) driver, which drives expression in the wing disc. Crosses were kept at 29°C to maximize the expression of the transgenes.

To visualize the ‘tracheal pattern’, the embryos carrying btlGal4 UAS-srcGFP (cell membrane staining) were stained for GFP. The bltGal4 in this combination also drives the other UAS transgenes.

Confocal images were acquired with a Leica TCS-SPE system. Images were post-processed with *ImageJ* and *Adobe Photoshop* and assembled using *Adobe Illustrator*.

### 2.5. NMR experiments   

An HSQC (heteronuclear single-quantum coherence) NMR experiment (eight scans and 128 increments in the indirect dimension) was recorded at 298 K on a Bruker Avance III 600 MHz spectrometer equipped with a *z*-pulse field gradient unit and a triple (^1^H, ^13^C, ^15^N) resonance probe head. The protein sample (0.7 m*M*) was equilibrated in 20 m*M* deuterated Tris–HCl, 150 m*M* NaCl buffer with 10% D_2_O and the pH adjusted to 7.5. The data were processed using the *XwinNMR* 3.5 software supplied with the Bruker NMR spectrometer.

### 2.6. Crystallization   

Initial crystallization conditions were identified from sparse-matrix screens. A series of gradient screens optimized the final condition and three-dimensional diffraction-quality crystals were finally grown in sitting drops at 293 K from 12%(*v*/*v*) 1,2-propanediol, 9%(*w*/*v*) PEG 20 000, 0.1 *M* glycine pH 9. The short protein construct crystallized in similar conditions.

### 2.7. Data collection   

Data were collected at 100 K from a monoclinic crystal using a PILATUS 6M detector on BL13-XALOC at the ALBA Synchrotron Light Source, Barcelona, Spain. 360 images were collected at a wavelength of 0.97949 Å with an exposure time of 1 s and an oscillation of 1°. Data were processed in the *XDS* suite (Kabsch, 2010 ▶) and validated using *POINTLESS* (Evans, 2006 ▶). Statistics of data-collection and model building are presented in Table 1 ▶.

### 2.8. Phasing, model building and refinement   

Structure solution and model refinement was performed with programs from the *PHENIX* suite (Adams *et al.*, 2010 ▶). A structural solution was obtained by molecular replacement with *Phaser* (McCoy *et al.*, 2007 ▶) in the *phenix.mr_rosetta* pipeline of programs (DiMaio *et al.*, 2009 ▶; Terwilliger *et al.*, 2012 ▶), using as a search model an ensemble constructed from the known structures of canonical MH2 domains: PDB entries 1khx (16% sequence identity; Wu *et al.*, 2001 ▶), 1mjs (16%; Qin *et al.*, 2002 ▶), 1khu (15%; Qin *et al.*, 2001 ▶), 1dd1 (15%; Qin *et al.*, 1999 ▶) and 3gmj (14%; Wang *et al.*, 2009 ▶).

Sequence alignment and selection of homologues was performed with *HHpred* (Söding *et al.*, 2005 ▶), and *Sculptor* (Bunkóczi & Read, 2011 ▶) was used to edit non-identical side chains (Schwarzenbacher *et al.*, 2004 ▶), subsequently superimposing the structures in *Ensembler*, which also removes nonconserved loops (http://www.phenix-online.org/documentation/reference/ensembler.html). The ensemble and the final model are superimposed and displayed in Supplementary Fig. S1.

Initial molecular-replacement solutions were rebuilt using *Rosetta* model completion and relaxation in *phenix.mr_rosetta*, and automated rebuilding yielded the best results starting from Smad2 MH2 (PDB entry 1khx), which resulted in a model with a free *R* factor of 30%.

Refinement was performed with *phenix.refine* (Afonine *et al.*, 2012 ▶) employing simulated-annealing and energy-minimization cycles. Initially tight geometry restraints were applied, which were released gradually as the *R*_work_ and *R*_free_ factors diverged. After several rounds of iterative refinement and model building in OMIT maps (Terwilliger *et al.*, 2008 ▶) and feature-enhanced maps (FEMs; Afonine *et al.*, 2015 ▶) in *Coot* (Emsley & Cowtan, 2004 ▶), density representing the N-terminal helix of the molecule could easily be identified in a difference electron-density map (*mF*_o_ − *DF*_c_). Iterative refinement and model building was continued until no significant improvement in *R* factors could be obtained. The refined model was validated using *MolProbity* (Chen *et al.*, 2010 ▶) and the *wwPDB Validation Server* (http://wwpdb-validation.wwpdb.org; Berman *et al.*, 2003 ▶). The Ramachandran plot is shown in Supplementary Fig. S2.

### 2.9. Structural analysis   

Structural superimposition was performed with the superpose algorithm in *PyMOL* (Schrödinger) based on the β-strand content of each structure as calculated by *DSSP* (Kabsch & Sander, 1983 ▶). Sequence alignments with the *EMBOSS Needle* algorithm returned values for sequence identity and similarity (Rice *et al.*, 2000 ▶).

## 3. Results and discussion   

### 3.1. Identification of a non-Smad MH2-like domain in protostomes   

The sequence identity of the Exp/Reb *Drosophila* proteins (also known as CG13188 and CG13183, respectively) is mostly restricted to the MH2 domain (∼30%). A comparison with Smads reveals a similarity about 16%, which is limited to the MH2 domain. This similarity suggests that the Exp/Reb and Smads proteins share the presence of the MH2 domain and that the Exp/Reb proteins may constitute a new protein family with a specialized function in a subset of metazoan phyla.

**Figure 1:**
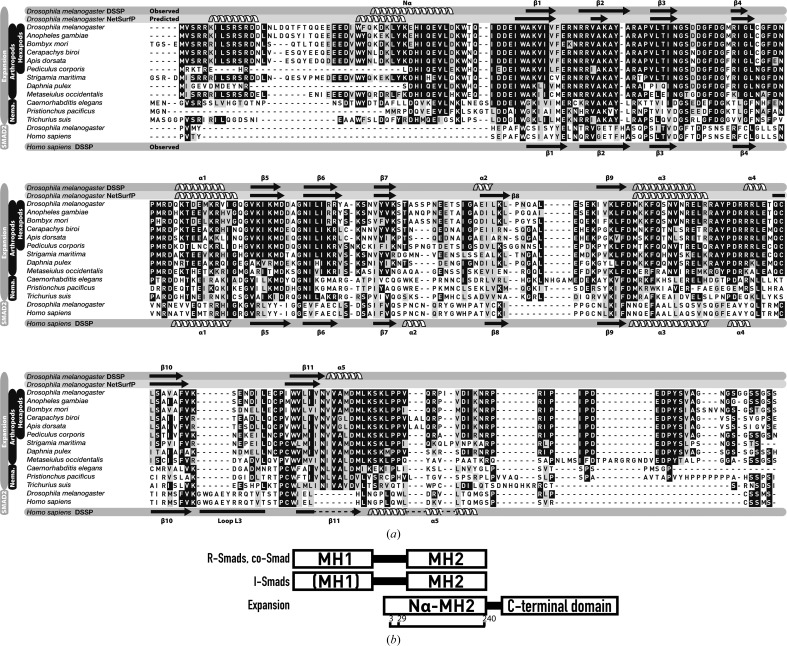
Sequence alignment of the N-terminal region of Expansion and Smad MH2 domains. (*a*) Sequence alignment of the N-terminal region of the *D. melanogaster* Expansion protein with orthologous proteins found in the Hexapoda, Arthopoda and Nematoda phyla. These sequences were obtained using *BLAST*. Using a *PSI-BLAST* search, we also detected sequence similarity to the MH2 domain of Smads. The *Homo sapiens* and *Drosophila* Smad2 MH2 domains are included in the alignment for comparison. Additional entries corresponding to uncharacterized proteins were not included in the alignment. A sequence alignment of the full-length proteins belonging to the Expansion family and a list of the entries included in the alignment are given as Supporting Information. The elements of secondary structure corresponding to the Expansion Nα-MH2 domain are highlighted at the top of the alignment, with the elements of secondary structure labelled. The results of the secondary-structure prediction used to guide us in the definition of the protein boundaries are shown below. For comparison, the secondary-structure elements corresponding to the human Smad2 domain are depicted below the alignment. (*b*) Schematic representation of the domain organization in Smad proteins and Expansion, indicating the boundaries of the prepared Nα-MH2 constructs.

To clarify this issue, we searched the Ensembl Metazoa database (http://metazoa.ensembl.org) using *PSI-BLAST* with the Expansion full-length sequence as the query. Our search retrieved two sets of matches. The first set reflects similarity to the entire query in nematodes, arthropods and other hexapods, suggesting the presence of orthologous proteins in these organisms. The second set reflects similarity of the N-terminal part of the sequence query to only the C-terminal part of Smads, specifically to the MH2 domain, as reported in the annotation of these proteins in the NCBI database. Remarkably, the similarity in the Exp/Reb subfamily covers an additional region of similarity preceding the canonical MH2 domain which is absent in the Smad sequences. The alignment comparing the sequences of the Exp/Reb domains with those of the MH2 domain of Smads is shown in Fig. 1 ▶(*a*).

Secondary-structure predictions using *NetSurfP* (Petersen *et al.*, 2009 ▶) corroborated the sequence similarity of the new domain to that of the Smad MH2 structure and indicated that the additional conserved region preceding the canonical MH2 fold might adopt a helical structure. Of the two predicted helices, the sequence that lies adjacent to the canonical MH2 domain is more conserved than the fragment predicted at the most N-terminal part of the protein. The prediction is depicted at a 0.4 level of probability in the sequence alignment (Fig. 1 ▶ *a*). A schematic representation of the domain organization of the Exp/Reb proteins and the similarity to Smads is shown in Fig. 1 ▶(*b*). An alignment based on the full sequence of the Expansion proteins is shown in Supplementary Fig. S3.

### 3.2. *Drosophila* Exp/Reb do not participate in the TGF-β pathway   

In addition to the structural work, we investigated the functional implication of this new family of proteins using cellular and genetic approaches in *Drosophila*. The TGF-β signalling pathway has been shown to play a key role in tracheal formation, specifying the most dorsal and ventral branches (Llimargas & Casanova, 1997 ▶; Ribeiro *et al.*, 2002 ▶; Vincent *et al.*, 1997 ▶). When the pathway is downregulated (by overexpressing the inhibitory Smad), the formation of dorsal and ventral tracheal branches is compromised. However, when the pathway is constitutively activated (by overexpressing a constitutively active Thick Veins receptor; Tkv^CA^) all branches migrate along the dorso–ventral axis (Supplementary Figs. S4*a*, S4*b* and S4*c*). We have observed that the pattern of dorsal and ventral branches was correct not only when Exp/Reb were downregulated using RNAi (Supplementary Figs. S4*d* and S4*e*) but also when both genes were removed using a combination of chromosome deficiencies that uncovers both of them (Supplementary Fig. S4*f*). In fact, we detected a completely different phenotype in loss-of-function conditions for these genes (Supplementary Figs. S4*d*, S4*e* and S4*f*), related to the accumulation of an apical chitinous extracellular matrix (aECM; Araújo *et al.*, 2005 ▶; Devine *et al.*, 2005 ▶; Tonning *et al.*, 2005 ▶; Moussian *et al.*, 2015 ▶). To further test any possible involvement of Exp/Reb in the TGF-β pathway, we also analyzed the phenotypes of their downregulation in the wing (also obtaining a negative effect) and compared them with the defects in the downregulation of the control Smad4/Medea. As depicted in Supplementary Figs. S4(*g*), S4(*h*) and S4(*i*), the detected phenotypes of Exp/Reb downregulation are very different from those of the control, confirming that Exp/Reb do not appreciably transduce TGF-β signals. Our results are consistent with recently reported experiments (Iordanou *et al.*, 2014 ▶) and suggest that this new family of proteins are involved in different functional pathways to the canonical TGF-β pathway.

Overall, these results support our hypothesis that the Exp/Reb proteins define a new family of proteins specific for protostomes that have the MH2 domain in common with Smads.

### 3.3. Recombinant expression and structural determination of the Nα-MH2 domain of an Exp/Reb protein   

#### 3.3.1. Protein expression, NMR and crystallographic screening   

Based on the sequence conservation and on the secondary-structure predictions, we selected two different domain boundaries for structural studies: a construct including the most conserved predicted helix (amino acids 29–240) and a second construct comprising nearly the full N-terminal region of the protein (amino acids 3–240) (Fig. 1 ▶ *b*). These two recombinant proteins were soluble and eluted as monomers in size-exclusion chromatography (Supplementary Fig. S5). According to NMR experiments the construct consisting of amino acids 29–240 was folded (Supplementary Fig. S6), and the initial crystallographic results were obtained using this construct. Since diffraction-quality crystals were also obtained from the larger construct, we focused the structural work on this construct consisting of amino acids 3–240. This would allow us to elucidate the role of the entire N-terminal region.

Diffraction data were collected from a monoclinic wedge-shaped crystal with approximate dimensions of 10 × 30 × 150 µm. The data were processed in space group *P*2_1_ to a maximum resolution of 1.6 Å. Matthews coefficient analysis indicated a solvent content of ∼27% for one protomer (∼28 kDa) in the asymmetric unit, which was consistent with the tight packing observed in the structure.

#### 3.3.2. Structural determination of the Nα-MH2 domain   

Owing to the anticipated structural resemblance of the new domain to the canonical Smad MH2 domain, we first attempted to solve the structure by conventional molecular replacement (MR) using *Phaser* (McCoy *et al.*, 2007 ▶) with several human Smad MH2 domains as search models (Table 2 ▶). Using this program we could identify a few potential MR solutions that could not be further refined, probably owing to the low sequence identity between the Expansion MH2 domain and human Smad MH2 (∼16%; Schwarzenbacher *et al.*, 2004 ▶). Among these solutions, the best was that obtained using the human Smad3 MH2 domain (PDB entry 1mjs). The selected solution (using *Phaser*) reported values of LLG = 56.1, TFZ = 4.3 and *R*_val_ = 58.5 at 3 Å resolution, and allowed us to partially trace the map. However, we were unable to perform rigid-body refinement as we could not improve the *R* factors beyond *R*_work_ = 0.5207 and *R*_free_ = 0.5215. Similar values were obtained for other resolution ranges.

Since this approach was unsuccessful, we decided to apply the *MR-Rosetta* algorithm, which has recently been demonstrated to facilitate molecular replacement in cases where only search models of low sequence identity are available. Using the methods compiled in the *MR-Rosetta* pipeline (DiMaio *et al.*, 2011 ▶; Terwilliger *et al.*, 2012 ▶) from the *PHENIX* package (Adams *et al.*, 2010 ▶) and an ensemble constructed from the known structures of canonical MH2 domains as a search model, a structural solution could be obtained. The ensemble and the final model are displayed in Supplementary Fig. S1. The data were cut following the recent recommendations by Karplus & Diederichs (2012 ▶) at a conservative resolution of CC_1/2_ > 0.5, which resulted in the data-collection and model-refinement statistics reported in Table 1 ▶. The high-quality diffraction data allowed the modelling of residues 27–236, with *R*_work_ = 0.1765 and *R*_free_ = 0.1977.

**Figure 2:**
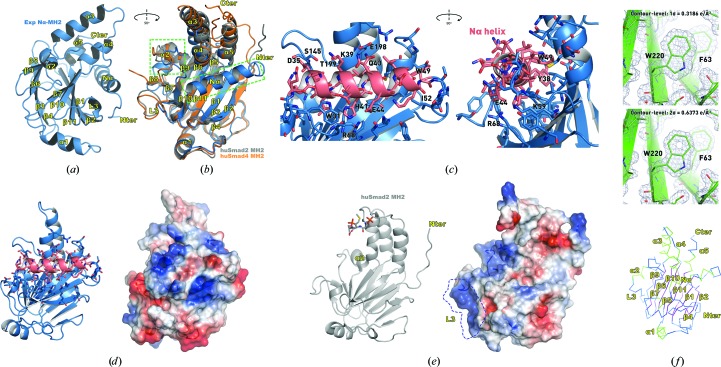
Structure of the Expansion Nα-MH2 domain and its comparison with the human Smad2 (huSmad2) and Smad4 (huSmad4) MH2 domains. (*a*) Refined structure of the Expansion Nα-MH2 domain including residues 27–236. The structure is displayed as a cartoon representation. Elements of secondary structure are labelled according to the Smad canonical MH2 fold, despite differences in the secondary-structure content in the N-terminus (the Nα helix present in Expansion) and in the region of α2 and β8+9. (*b*) Superposition of the huSmad2 MH2 domain (PDB entry 1mjs; grey), the human Smad4 MH2 domain (PDB entry 1dd1; orange) and the refined structure of the Nα-MH2 domain of Expansion (PDB entry 4r9p; blue). This view is rotated by 90° with respect to (*a*). (*c*) Close-up view of the Nα helix of the Expansion Nα-MH2 domain. The Nα helix (depicted in salmon) and the side chains that contribute to its packing are represented as sticks and labelled. (*d*) Cartoon and electrostatic potential surface distribution of the Expansion Nα-MH2 domain. (*e*) Same as in (*d*) for the huSmad2 MH2 domain (PDB entry 1khx). The L3 region required for phosphorylation recognition is highlighted. (*f*) The C^α^ trace of the Nα-MH2 domain and electron-density maps at contour levels of 1σ (0.3186 e Å^−3^) and 2σ (0.6372 e Å^−3^). All figures were prepared using *PyMOL* (Schrödinger).

As expected, the refined model revealed a tertiary structure with a striking resemblance to the canonical Smad MH2 fold (Figs. 2 ▶ *a* and 2 ▶ *b*). The structure comprises a β-sandwich core of twisted antiparallel β-sheets capped at one end by a three-helix bundle and at the other by a region containing an α-helix and several loops. In Smad MH2 domains this region is commonly referred to as the ‘loop–helix region’ (Shi *et al.*, 1997 ▶).

At a first glance, the most obvious structural difference when superimposed on Smad2 MH2 domains is the presence of the Nα helix, which is formed by the N-terminal residues 34–47 and covers one side of the core β-sandwich (Figs. 2 ▶ *c* and 2 ▶ *d*). This new element of secondary structure is named Nα to indicate its position at the N-terminus of the domain and, most importantly, to maintain the canonical nomenclature of MH2 domains (Fig. 2 ▶ *c*). A C^α^ trace of the Nα-MH2 domain and the electron-density map at contour levels of 1σ and 2σ are shown in Fig. 2 ▶(*f*). A few additional structural differences are observed in the length of the helices that form the helical bundle and in the area adjacent to the ‘loop–helix region’ comprised of helix α2, a loop and strand β8, referred to here as the ‘H2 region’ (Fig. 2 ▶ *b*, green rectangles). Also significant is the reduced length of the L3 loop that connects β10 and β11. This loop in Smad MH2 domains comprises an interaction motif for phosphorylated residues, and is fundamental to receptor binding and to the quaternary structure and function of the Smad proteins (Lo *et al.*, 1998 ▶). In Smad domains loop L3 comprises 17 residues, whereas in Expansion this loop is shorter (11 residues) and is very different in sequence (Fig. 1 ▶ *a*). To observe the effect of the differences in and around the L3 area, we have represented the surface charge distribution of the Expansion Nα-MH2 domain and that of Smad2 for comparison (Figs. 2 ▶ *d* and 2 ▶ *e*) and highlighted the presence of positively charged patches in the Smad2 MH2 domain that are absent in Expansion.

#### 3.3.3. The structure of the Nα-MH2 domain does not support the formation of homotrimers or heterotrimers   

**Figure 3:**
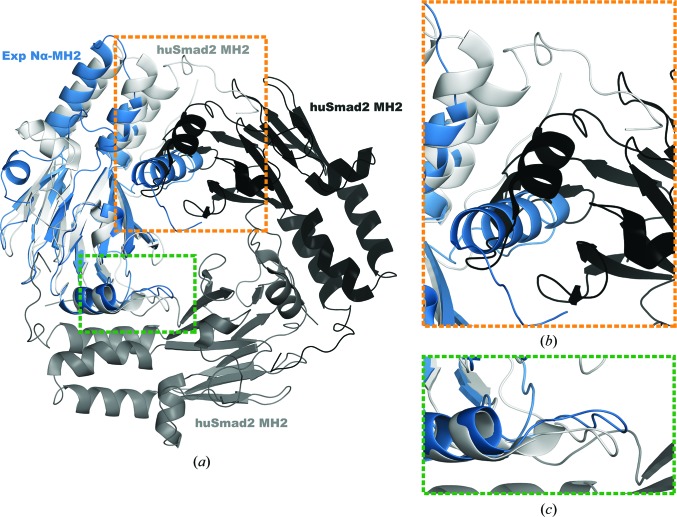
Comparison of the huSmad2 MH2 homotrimer with the Expansion Nα-MH2 domain. (*a*) Structural superposition of Expansion Nα-MH2 (blue) and the huSmad2 MH2 homotrimer. Smad2 MH2 domains in the trimer are represented as cartoons coloured three shades of grey to distinguish each monomer in the homotrimeric structure. The structures are superimposed using the β-sandwich (r.m.s.d. of 0.636 Å on 43 C^α^ atoms). All elements of secondary structure of Expansion and of one monomer of the human Smad2 trimer overlap, but the Nα helix of Expansion is superimposed on a loop of an adjacent MH2 domain of huSmad2. The superimposed Nα helix and the H2 region are marked with rectangles (gold and green, respectively). Detailed views of these regions are shown in (*b*) and (*c*).

The N-terminal helix observed in Expansion Nα-MH2 (Figs. 2 ▶ *a* and 2 ▶ *c*) is a new addition to the canonical MH2 fold characteristic of Smad proteins. This novel structural element packs against and interacts with the triple-helical bundle and covers the outer surface of the β-sandwich core, which represents the interface between adjacent monomers in the functional trimer of the Smad proteins. Previous results have suggested how cancer-derived mutations, which map to the same area in the Smad proteins, inhibit the formation of the functional heterotrimer (Shi *et al.*, 1997 ▶). When superposing the Nα-MH2 domain (shown in blue in Figs. 3 ▶ *a*, 3 ▶ *b* and 3 ▶ *c*) onto one monomer of the Smad2 MH2 homotrimer (shown in light grey), the Nα helix overlaps with the area occupied by the ‘loop–helix region’ of the adjacent monomer in the trimer (in the figure this second MH2 is shown in dark grey). This structural ‘clash’ most certainly compromises the formation of homotrimers by the Nα-MH2 domain in a manner similar to that observed for the Smad MH2 domains. Furthermore, it will also prevent the formation of heterotrimers with the MH2 domain of Smad proteins.

In Smad proteins, where the MH2 domain is located at the protein C-terminus (Fig. 1 ▶ *b*), the linker preceding the MH2 domain does not adopt a defined tertiary structure. However, in several structures of Smad complexes segments of the linker have been observed to adopt different conformations when folding upon interaction with other proteins (Aragón *et al.*, 2011 ▶, 2012 ▶; Shi & Massagué, 2003 ▶).

The secondary-structure predictions of the Expansion family of proteins suggested the presence of a second helix at the very N-terminus of the protein (Fig. 1 ▶ *a*). However, no electron density could be observed for this predicted helix, likely reflecting a degree of flexibility in this area. Patches of positive electron density observed in a difference-density map (*mF*_o_ − *DF*_c_) suggested that the region comprising the first 24 residues extends into a solvent channel of the crystal structure; however, any effort to improve the model in this region did not improve the refinement statistics and the model was therefore truncated at the N-terminus.

#### 3.3.4. Protein interactions in MH2 domains   

The divergent sequence of loop L3 in the Nα-MH2 domain indicates a significant difference in the function of the Expansion Nα-MH2 domain with respect to canonical Smad MH2 domains. In the TGF-β pathway signals are propagated through receptor activation of R-Smads by phosphorylation of the S-*X*-S motif at the C-terminus of the MH2 domain, leading to heterotrimer formation through the MH2 domain of two R-Smads with the common Smad4. Formation of the heterotrimer triggers the subsequent translocation of Smads to the nucleus. Mutations in the L3 loop of the Smad proteins abolish the formation of heterotrimers and hence signalling in the TGF-β pathway (Wu *et al.*, 2001 ▶). Whereas unphosphorylated Smad proteins are able to form dimers and trimers in a concentration-dependent manner (Shi & Massagué, 2003 ▶), the presence of the additional helix and the lack of the L3 loop in the Nα-MH2 domain could effectively prevent this domain from supporting the formation of similar dimers and trimers.

The Smad MH2 domain is commonly known to support protein interactions, and interestingly the overall composition of the Expansion Nα-MH2 domain remains largely the same despite the low degree of sequence identity. However, the H2 region that has been established as a protein–protein interaction site in Smad proteins (Qin *et al.*, 1999 ▶; Wu *et al.*, 2002 ▶) is completely different in the Expansion Nα-MH2 domain. In the refined model of the Nα-MH2 domain the β-sandwich core is comprised by two antiparallel β-sheets each having five strands, whereas in Smad MH2 the upper sheet comprises six strands (Figs. 2 ▶ *a* and 2 ▶ *b*). In the Expansion Nα-MH2 domain the region of amino acids 162–165 that would correspond to the β8 strand in Smads lacks a defined secondary structure; it instead bears characteristics of random coil similar to most of the adjacent H2 region, apart from helix α2. In Smad proteins the H2 region extends from strand β7 in the upper β-sheet, with helix α2 followed by a short loop that connects to strand β8 continuing to strand β9. In the Nα-MH2 domain strand β7 is followed by a large loop connected to a reoriented helix α2, followed by an extended region (corresponding to strand β8) continuing to strand β9 (Figs. 2 ▶ *a* and 2 ▶ *b*).

In the Smad proteins the H2 region and the β8 strand are implicated in protein–protein interactions by β-sheet augmentation, similar to common PDZ domains (Cowburn, 1997 ▶; Doyle *et al.*, 1996 ▶; Morais Cabral *et al.*, 1996 ▶; Schultz *et al.*, 1998 ▶), by annealing an additional β-strand to an existing β-sheet. This type of coordination is common in protein–protein interactions (Remaut & Waksman, 2006 ▶) and has been characterized structurally in three cases for Smad proteins. The structure of the isolated human Smad4 MH2 domain was solved from a construct with a protracted N-terminal boundary containing the first part of the linker region (for the ‘common’ Smad4 this is also known as the Smad-activation domain). As mentioned above, the structure revealed that part of the linker adopts an extended conformation and interacts with the H2 region by β-sheet augmentation (PDB entry 1dd1; Qin *et al.*, 1999 ▶). Similarly, the structure of Smad4 in complex with the repressor protein c-Ski (PDB entry 1mr1) also shows that the interaction occurs through β-sheet augmentation of strand β8 (Wu *et al.*, 2002 ▶). Moreover, the interaction between Smad2 and SARA (Smad anchor for receptor activation) has been characterized structurally and was also revealed to involve β-sheet augmentation, but not in the H2 region (PDB entry 1dev; Wu *et al.*, 2000 ▶). It is possible that secondary-structural changes induced by ligand binding could stabilize the β8 structure. Indeed, *NetSurfP* predicts the presence of strand β8 in Expansion, indicating (to some extent) an intrinsic folding property of this area. Of interest in the remodelling of the H2 region of the Expansion Nα-MH2 domain as a potential protein-interaction site is the consideration of the degree of specificity that this remodelling might provide.

#### 3.3.5. Structural classification in the Smad/FHA family   

Structural homology to the Smad MH2 domain has also been found in FHA domains and the C-terminal regulatory domain of IRF-3. Despite a complete absence of sequence conservation between these proteins, an evolutionary link has previously been suggested owing to the structural and distant functional similarities between the β-sandwiches in these proteins (Durocher *et al.*, 2000 ▶; Huse *et al.*, 2001 ▶; Takahasi *et al.*, 2003 ▶). Indeed, these proteins have all been classified into the same SCOP superfamily: the Smad/FHA domain. The superimposed structures of the human MDC1 FHA domain, the C-terminal regulatory domain of IRF-3 and the Expansion Nα-MH2 domain are shown in Supplementary Fig. S7. Since the MH2 domain of Smads is similar to the IRF-3 regulatory domain and to the FHA domain, the Nα-MH2 domain of Expansion is also a member of the same family of structures.

IRF proteins are only found in vertebrates, whereas the Smad MH2 domain and the FHA domain co-exist in metazoans and the FHA domain is also found in prokaryotes. Durocher *et al.* (2000 ▶) found the minimal β-sandwich of the FHA domain to comprise alternative protein-interaction sites, similar to those identified in the Smad MH2 domain, and Takahasi *et al.* (2003) speculated that the different flanking regions of the β-sandwich in the C-terminal regulatory domain of IRF-3 and Smad-MH2 developed on the β-sandwich scaffold of the FHA protein to facilitate phosphorylation signalling in higher organisms.

## 4. Conclusions   

We have determined the structure of the Nα-MH2 domain present in Expansion at 1.6 Å resolution. The addition of the N-terminal helix, the differences in the L3 loop and its lack of a role in the canonical TGF-β signalling pathway support the classification of Expansion as a new family of proteins that share the presence of the MH2 domain with Smads. Furthermore, the structural differences between the Smad MH2 and Expansion Nα-MH2 domains could have evolved to host a different range of protein-interaction partners, with implications for different cellular functions which apparently have been conserved in protostomes (Mollusca, Annelida and Arthropoda phyla).

For these reasons, we suggest that Expansion should not be termed a ‘Smad-like’ protein. Furthermore, we propose that the Smad/FHA family of structures should be ‘expanded’ to also include the Expansion Nα-MH2 domain.

## Supplementary Material

PDB reference: Expansion Nα-MH2, 4r9p

Supporting Information.. DOI: 10.1107/S1399004715001443/kw5112sup1.pdf

## Figures and Tables

**Table 1:** Data-collection and refinement statistics Values in parentheses are for the highest resolution shell.

Beamline	XALOC, ALBA
Wavelength ()	0.979
Resolution ()	31.31.59 (1.631.59)
Space group	*P*2_1_
Unit-cell parameters (, )	*a* = 36.8, *b* = 45.7, *c* = 54.2, = 90.00, = 93.31, = 90.00
Molecules per asymmetric unit	1
Unique reflections	23152 (2902)
Completeness (%)	95.5 (74.6)
*R*_meas_ †	0.071 (0.983)
*R*_merge_ ‡	0.069 (0.840)
Multiplicity	5.89 (3.5)
*I*/(*I*)	13.6 (1.1)
CC_1/2_ §	0.998 (0.506)
*B*_Wilson_ (^2^)	33.0
*R*_work_/*R*_free_ ¶	0.176 (0.323)/0.198 (0.350)
Average *B* factor (^2^)	
Protein	32.9
Water	38.9
R.m.s. deviation from ideal values	
Bond lengths ()	0.019
Bond angles ()	1.462
Ramachandran plot statistics (from *MolProbity* ††)	
Outliers (%)	0
Favoured (%)	98.2
*MolProbity* ‡‡ statistics	
Score	1.35
Clashscore	6.26
Rotamer outliers (%)	1.0
No. of atoms (non-H)	1740
No. of solvent molecules	75
PDB code	4r9p

† *R*_meas_ = 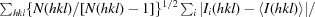 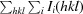.

‡ *R*_merge_ is defined according to Kabsch (2010 ▶).

§ CC_1/2_ is the correlation between intensities from random half data sets (Karplus Diederichs, 2012 ▶).

¶ *R*_free_ is the cross-validation *R* factor computed for a test set of reflections (5%) which were omitted from the refinement process.

†† R.m.s. deviation from ideal values in accordance with Engh Huber (2001 ▶).

‡‡ Chen *et al.* (2010 ▶).

**Table 2:** Human Smad MH2 domains used as search models for MR

PDB code	Description	Identity (%)
1khx	Human SMAD2 MH2 domain	16
1mjs	Human SMAD3 MH2 domain	16
1khu	Human SMAD1 MH2 domain	15
1dd1	Human SMAD4 MH2 domain	15
3gmj	*D. melanogaster* MAD MH2 domain	14
