# Supplementary material for: Structure of the N-terminal domain of the protein Expansion: an ‘Expansion’ to the Smad MH2 fold
Source: Acta Crystallogr D Biol Crystallogr. 2015 Mar 26;71(Pt 4):844–53. doi: 10.1107/S1399004715001443 (PMC4388265; doi:10.1107/S1399004715001443)
Supplement: Supplementary file 1 [file d-71-00844-sup1.pdf]

BIOLOGICAL CRYSTALLOGRAPHY

Volume 71 (2015)

Supporting information for article:

Structure of the N-terminal domain of the protein Expansion: an `Expansion' to the Smad MH2 fold

Mads Beich-Frandsen, Eric Aragón, Marta Llimargas, Jordi Benach, Antoni Riera, Joan Pous and Maria J. Macias

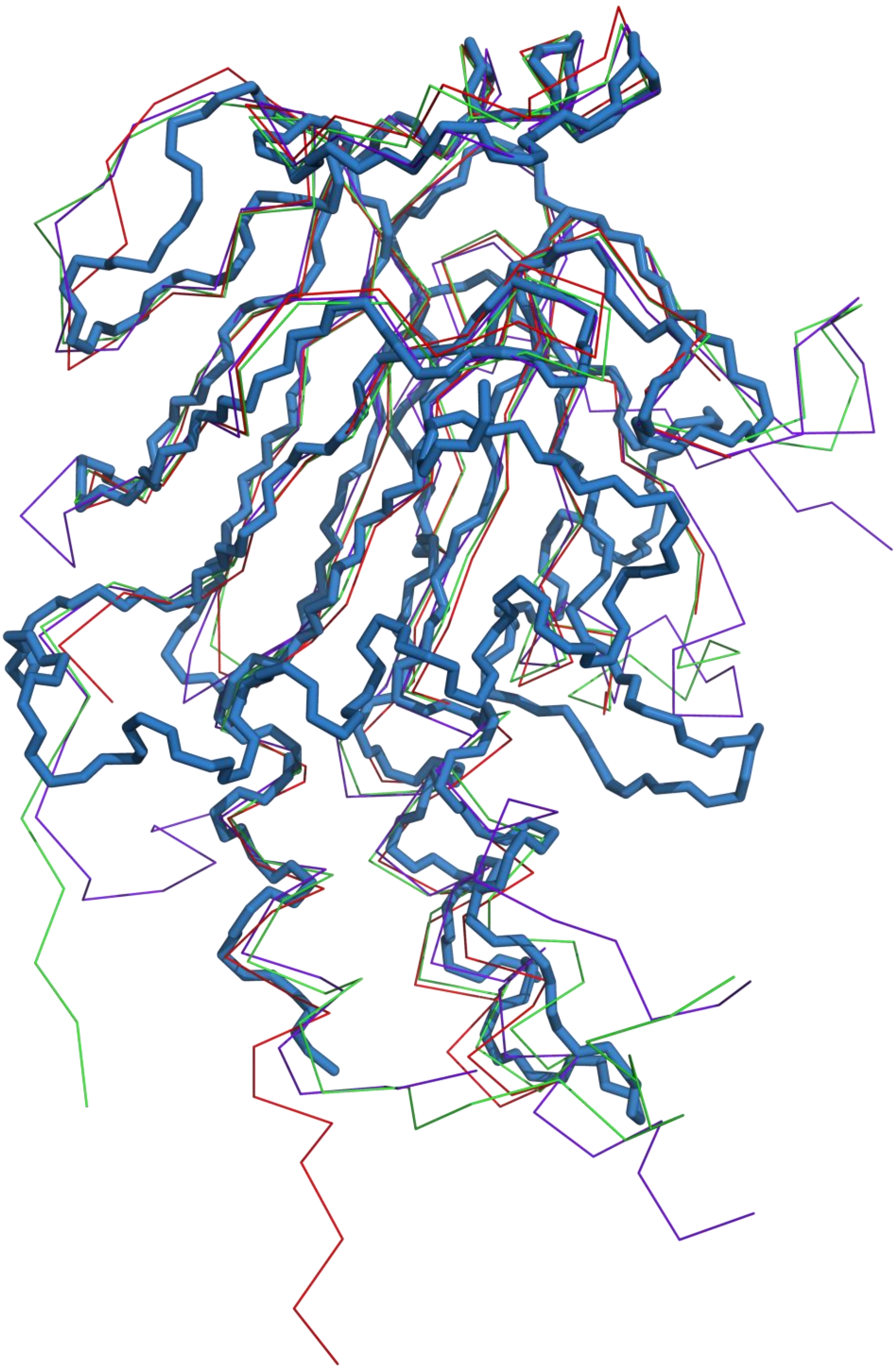

Figure S1 Superposition of the refined Expansion Nα-MH2 to the ensemble used in MR. The superposition shows the structural similarity of the refined structure (blue) with some of the structures in the ensemble used for molecular replacement., Smad2 (PDB-Code 1KHX, green) Smad3 (PDB-Code 1MJS, red) and Smad4 (PDB-Code 1DD1, purple).

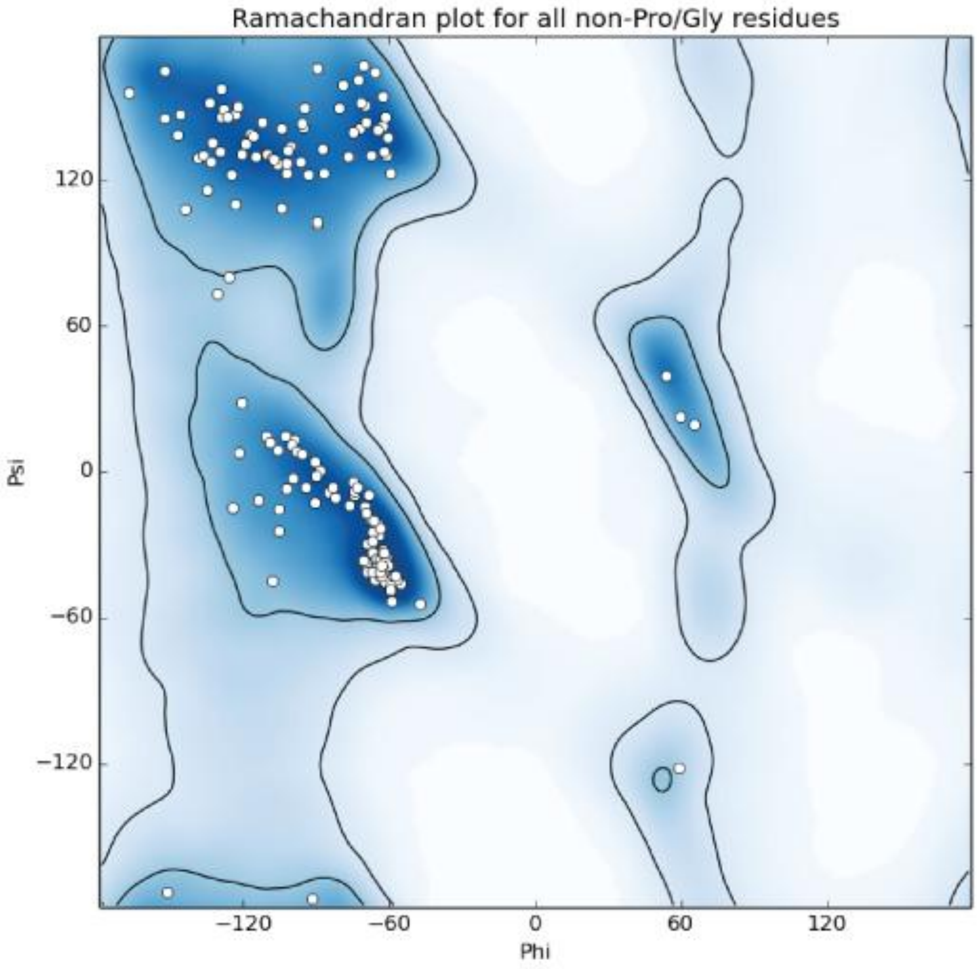

Figure S2 Ramachandran plot of the Expansion Nα-MH2 domain.

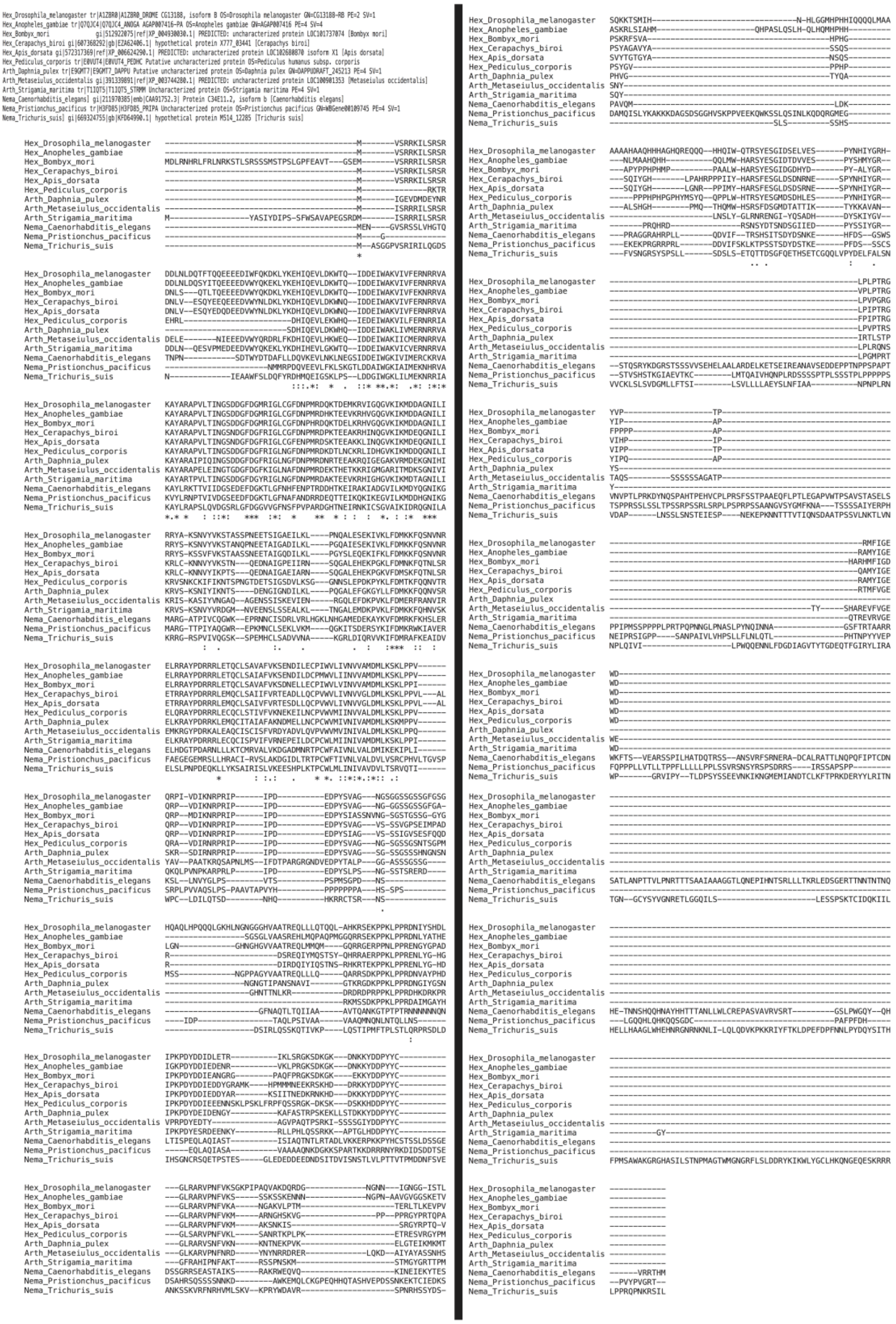

Figure S3 Sequence alignment of Expansion proteins. Sequence alignment of the full length proteins including *Drosophila melanogaster* Expansion, with the orthologous proteins found in Hexapoda, Arthopoda and Nematoda phyla.

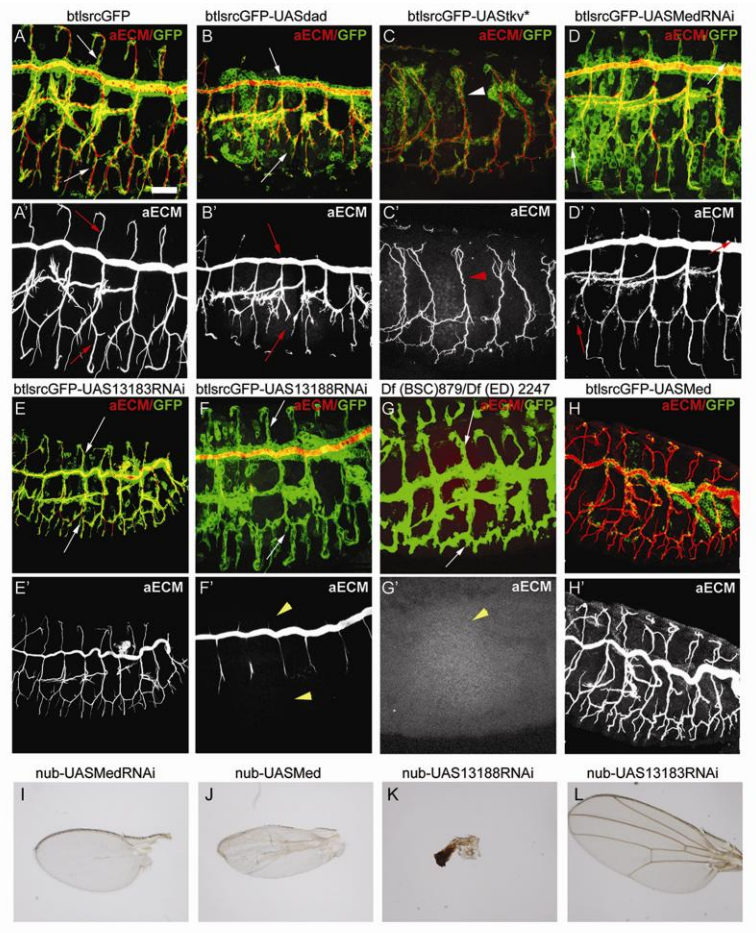

Figure S4 Comparison of TGF-β and Exp/Reb phenotypes. (a-h) Projections of confocal sections showing the tracheal metameres of embryos at stage 15 in lateral views. Scale bar 25 µm. (i-l) Bright field images showing dissected adult wings. In the wild type, the tracheal tree is formed by a thick longitudinal trunk and several dorsal and ventral branches (arrows in (a)). The pattern can be visualised by using cell markers (btlsrcGFP, green in all images) and markers for the luminal aECM (in red or black and white). When the Dpp pathway is downregulated the dorsal and ventral branches are compromised (see missing branches marked with arrows in (b) but the aECM is present. When the pathway is constitutively activated the branching pattern is affected (see all branches extending dorso-ventrally, arrowhead in (c) but the aECM is correct (c). The downregulation of Med gives rise to occasional and mild defects of dorsal and ventral branching (arrows in (d)), while its overexpression (h) does not produce detectable defects. When CG13188 and CG13183 are downregulated the branching pattern (visualised by the cellular marker in green) is correct (arrows in (e,f,g)), but the aECM is affected (arrowheads in (f',g')). The downregulation or overexpression of Med or CG13188 and CG13183 in the wing imaginal disc gives rise to very different phenotypes.

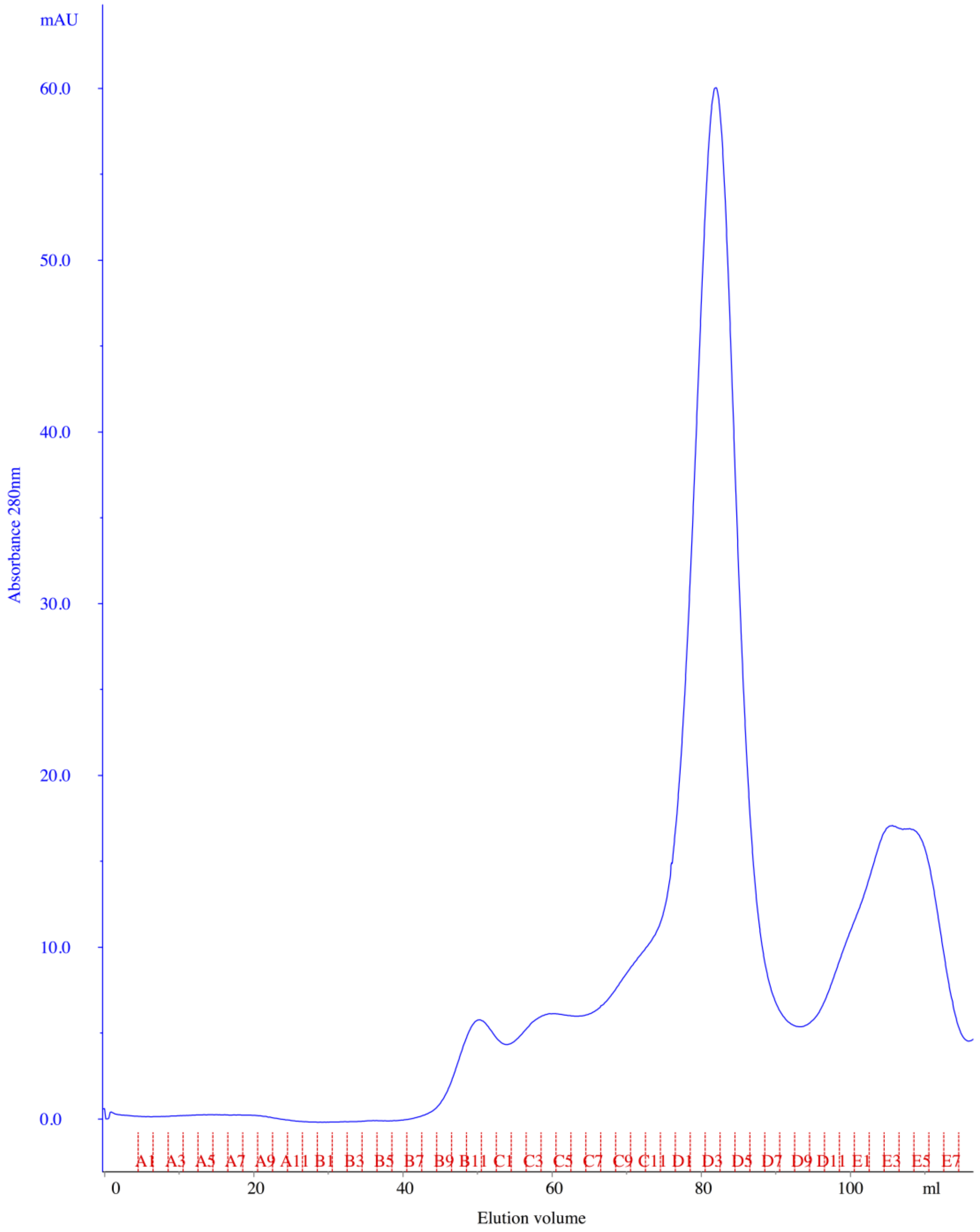

Figure S5 Size exclusion chromatography profile of the Expansion Nα-MH2 domain. The profile shows a main peak, indicating that the predominant form is monomeric. The elution volume corresponded to the molecular weight of a monomer. This was obtained using a Superdex 200, 10/300 column.

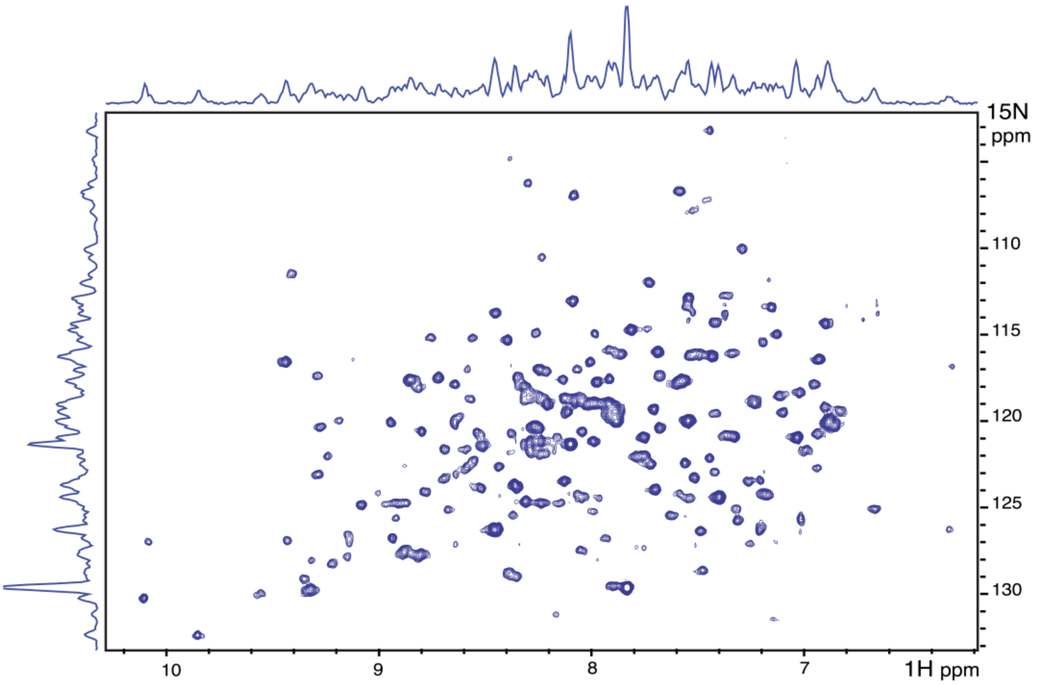

Figure S6 15NHSQC of the Expansion Nα-MH2 domain. This experiment was obtained using the 25-236 residues fragment. The $^{15}N^{1}H$-HSQC experiment shows a dispersion characteristic of a well-folded protein.

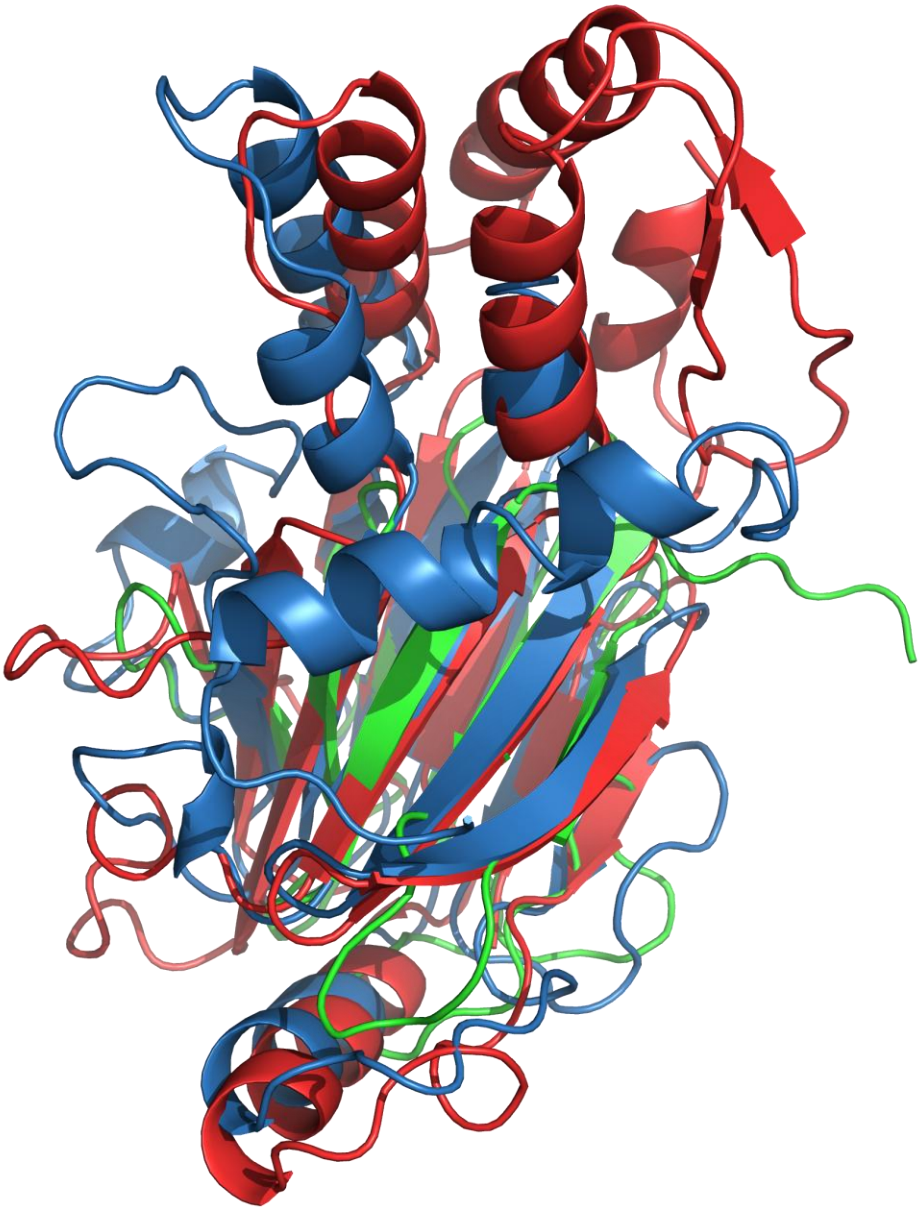

Figure S7 Comparison of Nα-MH2 domain and FHA domains. Cartoon representation of the Expansion Nα-MH2 domain (blue) superimposed to the human MDC1 FHA domain (green, PDB-code 3UNN) and human IRF3 FHA domain (red, PDB code 1J2F)
